# Increasing Completion Rate of an M4 Emergency Medicine Student End-of-Shift Evaluation Using a Mobile Electronic Platform and Real-Time Completion

**DOI:** 10.5811/westjem.2016.5.29384

**Published:** 2016-06-16

**Authors:** Matthew C. Tews, Robert W. Treat, Maxwell Nanes

**Affiliations:** * Medical College of Wisconsin, Department of Emergency Medicine, Milwaukee, Wisconsin; † ProHealth Waukesha Memorial Hospital, Emergency Medicine Associates of Waukesha, LLC, Waukesha, Wisconsin

## Abstract

**Introduction:**

Medical students on an emergency medicine rotation are traditionally evaluated at the end of each shift with paper-based forms, and data are often missing due to forms not being turned in or completed. Because students’ grades depend on these evaluations, change was needed to increase form rate of return. We analyzed a new electronic evaluation form and modified completion process to determine if it would increase the completion rate without altering how faculty scored student performance.

**Methods:**

During fall 2013, 29 faculty completed paper N=339 evaluations consisting of seven competencies for 33 students. In fall 2014, an electronic evaluation form with the same competencies was designed using an electronic platform and completed N=319 times by 27 faculty using 25 students’ electronic devices. Feedback checkboxes were added to facilitate collection of common comments. Data was analyzed with IBM® SPSS® 21.0 using multi-factor analysis of variance with the students’ global rating (GR) as an outcome. Inter-item reliability was determined with Cronbach alpha.

**Results:**

There was a significantly higher completion rate (p=0.001) of 98% electronic vs. 69% paper forms, lower (p=0.001) missed GR rate (1% electronic. vs 12% paper), and higher mean scores (p=0.001) for the GR with the electronic (7.0±1.1) vs. paper (6.8±1.2) form. Feedback checkboxes were completed on every form. The inter-item reliability for electronic and paper forms was each alpha=0.95.

**Conclusion:**

The use of a new electronic form and modified completion process for evaluating students at the end of shift demonstrated a higher faculty completion rate, a lower missed data rate, a higher global rating and consistent collection of common feedback. The use of the electronic form and the process for obtaining the information made our end-of-shift evaluation process for students more reliable and provided more accurate, up-to-date information for student feedback and when determining student grades.

## INTRODUCTION

The end-of-shift evaluation is a common method used to assess medical student clinical performance in emergency medicine (EM).^1^ Evaluation forms are completed at the end of each shift; they form the basis for formative feedback and contribute to the summative portion of a medical student’s rotation grade. End-of-shift forms used for formative feedback give students an opportunity to improve their performance on subsequent shifts and provide mid-rotation feedback for behavioral changes prior to their final evaluation.^2^ Nearly 80% of EM programs use an end-of-shift form, and these are commonly compiled into a cumulative, summative score that accounts for an average two-thirds of a student’s final grade.^1^ Despite the implications for student grades, there is a wide variety of methods used to obtain these forms and completion rates are highly variable.^3^–^5^

Challenges in the use of end-of-shift evaluations in EM include students working with different faculty throughout a given rotation, the wide variety of clinical experiences, and the variable experience and interest of students in the specialty.^6^ All of these challenges increase the difficulty of obtaining the forms and assessing the learner’s progress over time.^7^,^8^ Even with the widespread adoption of electronic technology in the medical profession, evaluative forms still remain paper-based and this creates additional logistical problems with the collection of data.^5^ The literature is sparse with reports on how to improve completion rates of these forms. No information could be found that describes using a mobile platform specifically for increasing completion rates of end-of-shift evaluations in EM.

The Department of Emergency Medicine at the Medical College of Wisconsin had used a paper-based end-of-shift evaluation form for fourth-year medical students until June 2014. The scores from these forms determined a significant portion of the students’ final rotation grade, but collecting completed forms was challenging. Forms would either be handed in incomplete or frequently would be misplaced. Consequently, we identified the need to more securely collect end-of-shift evaluations and improve their completion rate. We created an electronic student evaluation form that could be used on the students’ mobile electronic devices to replace the existing paper-based form and rolled out the new process in our department. The purpose of this study was to determine if the implementation of a new electronic evaluation form and modified completion process would increase the faculty completion rate without altering how faculty scored student performance by comparison with the previously used paper-based form.

## METHODS

The Medical College of Wisconsin has fourth-year medical students rotate at Froedtert Hospital, the primary clinical site for our EM residency program and a Level I trauma center with over 65,000 annual visits. Each month, up to 10 fourth-year students participate in an elective month-long rotation. Students are evaluated at the end of each shift using seven competencies and an overall global rating (Table 1) that aligns with the institution’s end-of-rotation global competencies for fourth-year students. During each shift, students are paired with both residents and faculty for their entire shift, but faculty provide the primary source of evaluation scores of the students’ daily shift performance. Students could opt out of having their data used for the study at any time, but the evaluations were a required component for determining clinical performance and the final rotation grade. This study was classified as exempt by the institutional review board at the Medical College of Wisconsin.

### Data Collection

At their rotation orientation the students were provided the end-of-shift form – paper for 2013–14 and the electronic link for 2014–15 – and instructed to ask the faculty to complete the respective form near the end of each shift. Faculty scored each student’s performance using an identical scale from “1” (lowest rating) to “9” (highest rating) for each competency on either form. The rotation form uses the same nine-point scale as our institution uses for the final rotation competencies, which allowed easy translation of the end-of-shift data into scores used with the institution’s previously existing end-of-rotation format.

### Paper Form

In the fall of 2013 faculty completed the paper form at the end-of-shift per our existing process. Students would complete the fields that included their name, date and shift time and then give the form to their faculty near the end of a shift. The form included competency items, an area for comments and a signature area for the faculty. Faculty members were responsible for placing the completed and signed form into a dedicated box located in the emergency department (ED), which was then emptied approximately once per week by our student coordinator. The form was usually completed by faculty before leaving their shift, but in some cases forms left the department with faculty and were intended to be completed in the following days or were accidentally left in the ED. Once a form was recognized as being missing, it would be placed in the faculty’s mailbox or attached via a reminder email. This resulted in variable response rate, and therefore a final analysis at the end of the month revealed which forms still needed completion. The data on the collected forms were transferred manually into a Microsoft Excel spreadsheet for data warehousing and subsequent analysis.

### Electronic Form

At the beginning of the 2014–15 academic year, an online survey program was used to design an electronically accessed and submitted student end-of-shift evaluation form. All competencies and scales from the paper form were transcribed verbatim into the electronic format (Table 1). The form was compatible with mobile and desktop platforms. The consistent use of electronic devices by all students was possible because each student was required by the medical school to have an electronic tablet device before they entered into their clinical rotations.

Prior to implementing the form, faculty were provided an overview of the challenges with the current form and the plan for switching to an electronic format. The new system for collection was described by email and in a four-minute podcast that demonstrated the completion of the electronic form. This was followed by periodic communication and discussion in faculty meetings updating them on the form completion rates.

The process for completing the form required that students first complete basic demographic and shift information, including their name, shift type, date and faculty name on the form. Students then gave the device with the open form to the faculty who completed the components and submitted the form, subsequently handing the device back to the student. Submission of the form was not possible until required fields were completed. Faculty “signed” the electronic form by using a five-digit individual identifier. If this identifier was missing or incorrect the evaluation would be considered invalid. Once submitted, the data were automatically uploaded to the survey database and were immediately available to be viewed by the student coordinator and downloaded into a spreadsheet for analysis.

### Comments

Using qualitative content analysis, we analyzed the comments written on the paper forms for the fall 2013–14 to determine common themes. Beginning with an inductive process, two authors used open coding to determine initial themes. Seven themes emerged based on the words used most frequently, both positively and negatively. After determining these themes, the authors used a deductive approach to revise the categories, which were then inserted into the electronic form where each theme was an individual checkbox item. These checkboxes were further separated by what the student did well and what the student needed to work on (Table 4). An area for additional comments was included.

### Outcomes

The primary outcome of this study was the comparison of overall faculty completion rate of both the paper and electronic formats in the fall cohorts for 2013–14 and 2014–15, respectively. Each shift was expected to result in one evaluation per student, and the number of evaluations expected was compared to the actual number of evaluations completed. Secondary outcomes included comparing the paper and electronic forms for the number of missing data points for the seven competencies and global rating, whether or not faculty members would score students consistently using the global rating and the frequency and usage of feedback checkbox and free text comments. Tertiary outcomes included analyzing the electronically submitted data between fall and winter student cohorts of 2014–15 since student interest in securing an EM residency is much higher in the fall.

We analyzed all data with IBM^®^ SPSS^®^ 21.0. Pearson chi-square tests assessed differences in completion rates of the different forms. We used analysis of variance (ANOVA) to determine differences in competencies and global ratings due to evaluation platform (paper or electronic) and interest in EM as a specialty (fall vs winter). Inter-item reliability of the seven competencies was determined with Cronbach alpha.

## RESULTS

Table 2 reports the descriptive statistics and Table 3 reports the mean competency scores and differences for the outcomes from the paper and electronic forms.

The paper form for fall 2013–14 was completed by 29 faculty N=339 times for 33 students. The electronic form for fall 2014–15 was completed by 27 faculty N=319 times for 25 students. Of these 319 evaluations, faculty completed 283 forms (89%) on tablets, 24 (8%) on a desktop computer and 12 (4%) on a smartphone. The overall completion rate was significantly higher (p=0.001) for the electronic form (98%) than for the paper form (69%).

The number of missing global ratings demonstrated a statistically significant improvement (p=0.001) from 39 (12%) on the paper form to near zero (0.6%) on the electronic form.

Overall, faculty scored students statistically higher for the global rating section on the electronic form versus the paper form (p=0.001).

Free text comments were documented on 89% of the written forms and 52% of the electronic forms. Feedback checkboxes for what the student did well were completed on 100% of the electronic forms and had a 90% completion rate for what the student needed to work on (10% documented no deficiencies for the shift). Table 4 shows the frequency of themes on the paper and electronic forms.

The winter electronic form cohort for 2014–15 was compared to the fall electronic 2014–15 cohort and demonstrated no significant differences for completion rate (p=0.872), missing global rating scores (p=0.872) and mean global rating scores (p=1.00).

To determine the internal consistency of scoring the seven competencies between the paper and electronic forms, we calculated Cronbach-alpha values and reported them to be 0.95 for data collected from both forms.

## DISCUSSION

The use of end-of-shift evaluation forms in EM is commonplace, yet there are challenges to consistently collecting completed forms. Paper forms can easily be left in the ED, misplaced, accidentally discarded, or found after students’ grades are submitted. Most studies in other specialties have reported the use of “encounter cards” to increase student satisfaction and improve the amount of formative feedback given during rotations, but the use of end-of-shift evaluations in EM has not been well described.^9^–^11^

Despite the regular use of electronic platforms in education, there are few descriptions in the literature of current practices that have successfully been implemented to increase evaluation completion rates. Manchester Medical School described the deployment of iPads to all of their students and successfully implementing the use of eForms to replace their paper-based systems, and reported that it was a more efficient system.^12^ Paukert, et al, described using encounter cards to improve student satisfaction with verbal and written feedback on a surgery clerkship.^13^ Bandiera and Lendrum examined the use of daily encounter cards based on the 2005 CanMEDS competency framework and found that EM teachers provided specific competency-based feedback after individual shifts, which when compiled covered the breadth of the competencies.^3^,^14^ To the best of our knowledge, no studies have compared the use of an electronic mobile platform to paper forms with the goal of increasing the collection of shift evaluations for summative purposes.

In our study, we identified the need to improve our end-of-shift evaluation completion rate and chose to use a new electronic platform with a modified completion process as our primary outcome. The design of the form and data collection were simple, and our electronic form mirrored our paper form with the exception of the feedback checkboxes. While the use of an electronic mobile platform was the main contributor to the success of the study, we believe that the process used to collect these electronic end-of-shift forms had the strongest impact on the improved rate of form return. This is likely related to several factors that influenced our outcomes.

First, the faculty used students’ devices to complete and submit the form. The expectation was that by providing their device to the faculty to use to complete the form, the student would get it back immediately. Visiting students from other institutions always had the option to open the form on any handheld device (including smartphones) or any available desktop computer in the department. Surprisingly, 11% of the forms were completed in one of these two alternate ways.

Second, the electronic format allowed the designers to indicate required fields. The evaluator would be redirected to the incomplete sections if submission of the form was attempted before all sections were complete. This required the evaluator to complete the form in its entirety “on the spot” once started, and forms could not be saved or completed at a later date. This completely resolved the missing data issue on submitted forms. The “missing” global rating scores we reported were actually a result of faculty marking “N/A” and choosing not to provide a score, although it is not known why. There were no actual missing data points for any submitted forms.

Finally, there were planned and purposeful communications with the faculty as we implemented the change in the platform and process of form completion. Creating awareness is an important component of change management, but was not the only factor that made our overall process successful. While we had considered steps to increase the response rate with the paper forms, doing so would have still resulted in the same limitations and challenges that come with their use. The change to the platform and process of form completion and collection turned out to be more valuable than just simply increasing completion of paper forms.

The secondary outcomes examined changes in the number of missing data points, whether or not faculty members would score students consistently with either the electronic or paper format and frequency and usage of feedback checkbox and free text comments. There were no missing data points for submitted forms in the electronic format, which yielded a significant improvement over the paper format. Additionally, it turns out that faculty scored students marginally higher using the electronic form, even though the content and organization of the form was the same between the paper and electronic form, using the same competencies, scales, and labels. We suspect that the reason for this was that, unlike the paper form in which it was not uncommon to have missing forms, all electronic forms that were started were completed and therefore represented a near-complete data set. While difficult to prove, the higher scores were therefore likely a more accurate representation of their performance, as opposed to inflation of the students’ grades by simply using the electronic platform, although inflation was still possible, but for unknown reasons.

The feedback checkboxes allowed faculty to click on the most common themes traditionally written about in the paper comments section. Organizing the themes into checkboxes allowed faculty to focus their comments on other areas that they felt were important in the free text section. We found it encouraging that half of the time for the electronic form faculty decided to type additional comments above and beyond the feedback checkboxes. While the frequency of free text comments was greater on the paper forms, using the electronic checkboxes allowed a simple method for identifying patterns of feedback to provide students across multiple shifts and over the course of the rotation, such as the need to work on their differential diagnosis. This was viewed as an improvement in our feedback process for our mid-rotation feedback sessions, since many paper forms were not even available to review with the students. We now have up-to-date qualitative and quantitative data available to share with students at any point.

Tertiary outcomes examined faculty patterns of scoring for students’ interest in securing an EM residency versus those who were not (fall versus winter 2014 cohorts, respectively). Using the electronic form, there was no difference in how faculty scored students, regardless of their interest in EM. This demonstrates consistency in evaluating students and lack of a bias favoring either group of students.

In the process of using this form, we learned there were a few drawbacks to using the electronic format with the students’ mobile device based on feedback from our faculty. First, the faculty identified that it was more difficult and time consuming to type comments into a mobile electronic device than write them on paper. However, the paper form had posed challenges with the comments section including being able to read what was written, receiving generic feedback such as “good job” and the lack of comments being written at all. Second, faculty commented that some students tended to wait near them as they completed the form, which the faculty felt was awkward. This was an unintended consequence of having the students use their own devices to allow faculty to complete the form, and was not monitored in our study.

## LIMITATIONS

This was a single institution with a single evaluation form and a limited number of students. However, even though each institution or department develops their own form and method of data collection, the overall process of increasing our completion rate was effective and used a commercially available product that was easy to use. With the dearth of free and for-cost survey platforms available, the user can review and choose the one that works best for them and work to make it fit their needs.

The use of checkboxes provided an efficient way for faculty complete the form and a more consistent availability of feedback for students and faculty at mid-rotation feedback sessions. However, we do not have data to suggest whether this method was adequate for students’ feedback needs compared to written or typed comments.

The use of the form for formative purposes was not a part of our study. Ideally, we would have had faculty review the students’ performance using the form as a guide. By doing this, it may have increased the completion rate further and provided students a learning opportunity with the feedback they would receive at the end of a shift.

## CONCLUSION

The use of a new electronic form and modified process for evaluating students at the end of shift demonstrated a higher faculty completion rate, a lower missed data rate, a higher global rating and consistent collection of commonly used feedback. Switching to electronic end-of-shift evaluations improved the evaluation process for faculty and students and has become more reliable, providing more accurate, up-to-date information for student feedback and to determine student grades, which continues to date. The use of an electronic form with our process has the potential to provide a way for others to improve their end-of-shift evaluation completion rate.

## Figures and Tables

**Table 1:** End-of-shift assessment components.

Communication: interpersonal skills/teamwork
Communication skills: presentation/documentation
History & physical exam skills/time management
Technical procedures: list type and proficiency
Patient care: medical problem-solving and decision-making
Patient care: management
Professional behavior and development
Overall

**Table 2:** Descriptive statistics of evaluation forms.

Form	Alpha	# Evaluations completed	% Completion	# Faculty	# Students	Mean evaluations completed	Missing global rating
Paper - EM	0.95	339	69	29	33	10.2/14	39
Electronic - EM cohort	0.95	319	98	27	25	12.8/13	2
Electronic - non-EM cohort	0.94	131	92	23	11	11.9/13	1

*EM,* emergency medicine

**Table 3:** Mean competency scores for M-4 emergency medicine students (N=787).

Competency	Mean (SD)			Statistical significance of pairwise differences between groups	

	Paper	Electronic			
		
	Fall 13/14 (Group 1)	Fall 14/15 (Group 2)	Win 14/15 (Group 3)	1/2	2/3
Communication: interpersonal skills/teamwork	6.8 (1.2)	7.2 (1.1)	7.1 (1.0)	0.001	1.00
Communication skills: presentation/documentation	6.7 (1.3)	7.0 (1.2)	7.0 (1.1)	0.002	1.00
History and physical exam skills/time management	6.5 (1.2)	6.8 (1.2)	6.9 (1.0)	0.003	1.00
Technical procedures: list type and proficiency	6.9 (1.1)	7.3 (1.2)	7.5 (1.1)	0.020	1.00
Patient care: medical problem-solving and decision-making	6.7 (1.2)	6.8 (1.2)	6.8 (1.0)	0.289	1.00
Patient care: management	6.7 (1.2)	6.9 (1.2)	6.8 (1.0)	0.128	1.00
Professional behavior and development	7.2 (1.1)	7.7 (1.0)	7.7 (0.9)	0.001	1.00
Global rating	6.8 (1.2)	7.0 (1.1)	7.1 (1.0)	0.018	1.00

**Table 4:** Frequency and percentages of comments on paper and electronic forms.

	Paper free text	Electronic feedback checkboxes	
		
Item	Frequency (%) (N=339 evaluations)	Student did well: frequency (%) (N=319 evaluations)	Student needs to work on: frequency (%) (N=319 evaluations)
Proactive/motivated	63 (19)	191 (60)	37 (12)
Personal interactive skills	56 (17)	271 (85)	8 (3)
Case presentations	54 (16)	129 (40)	129 (40)
Differential diagnosis	52 (15)	62 (19)	163 (51)
Teamwork	47 (14)	191 (60)	25 (8)
Medical knowledge	44 (13)	100 (31)	98 (31)
Time management	32 (9)	128 (40)	67 (21)
Total	348	1072	527

## References

[1] Khandelwal S, Way DP, Wald DA (2014). State of Undergraduate Education in Emergency Medicine: A National Survey of Clerkship Directors. Acad Emerg Med.

[2] Coates WC (2004). An educator’s guide to teaching emergency medicine to medical students. Acad Emerg Med.

[3] Kogan JR, Shea JA (2008). Implementing feedback cards in core clerkships. Med Educ.

[4] Kim S, Kogan JR, Bellini LM (2005). A randomized-controlled study of encounter cards to improve oral case presentation skills of medical students. J Gen Intern Med.

[5] Bandiera G, Lendrum D (2008). Daily encounter cards facilitate competency-based feedback while leniency bias persists. CJEM.

[6] Bandiera G, Lee S, Tiberius R (2005). Creating effective learning in today’s emergency departments: how accomplished teachers get it done. Ann Emerg Med.

[7] Santen SA, Peterson WJ, Khandelwal S (2014). Medical student milestones in emergency medicine. Acad Emerg Med.

[8] Greenberg LW (2004). Medical students’ perceptions of feedback in a busy ambulatory setting: a descriptive study using a clinical encounter card. South Med J.

[9] Richards ML, Paukert JL, Downing SM (2007). Reliability and usefulness of clinical encounter cards for a third-year surgical clerkship. J Surg Res.

[10] Bennett AJ, Goldenhar LM, Stanford K (2006). Utilization of a formative evaluation card in a psychiatry clerkship. Acad Psychiatry.

[11] Bernard AW, Kman NE, Khandelwal S (2011). Feedback in the emergency medicine clerkship. West J Emerg Med.

[12] Mooney JS, Cappelli T, Byrne-Davis L (2014). How we developed eForms: an electronic form and data capture tool to support assessment in mobile medical education. Med Teach.

[13] Paukert JL, Richards ML, Olney C (2002). An encounter card system for increasing feedback to students. Am J Surg.

[14] Frank JR The CanMEDS 2005 Physician Competency Framework.

